# Shedding light on the calculation of electrode electroactive area and heterogeneous electron transfer rate constants at graphite screen-printed electrodes

**DOI:** 10.1007/s00604-023-05832-w

**Published:** 2023-06-07

**Authors:** Maria G. Trachioti, Alexandros Ch. Lazanas, Mamas I. Prodromidis

**Affiliations:** Department of Chemistry, University of Ioannina, 45 110 Ioannina, Greece

**Keywords:** Electrode electroactive area, Heterogeneous electron transfer rate constants, Screen-printed electrodes, Chronocoulometry, Voltammetry, Electrochemical impedance spectroscopy

## Abstract

We present in detail the most known and commonly used methods for the calculation of electrode electroactive area ($A$) and heterogeneous electron transfer rate constants (${k}^{0}$). The correct procedure for the calculation of these parameters is often disregarded due to either lack of a minimum theoretical background or oversimplification of each method’s limitations and prerequisites. The aim of this work is to provide the theoretical background as well as a detailed guide for the implementation of these measurements by impressing upon the electrochemists the parameters that need to be considered so that the obtained results are safe and useful. Using graphite screen-printed electrodes, $A$, and ${k}^{0}$ were calculated with different methods and techniques. Data are compared and discussed.

**Graphical Abstract:**

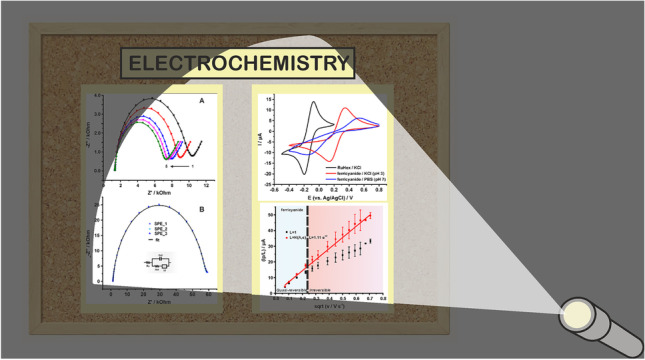

## Theoretical background

### *Calculation of electrode electroactive area (*$A$*)*

An important parameter to consider when evaluating the response of an electrochemical system is the electroactive area of the working electrode as the peak current (${I}_{p}$) is dependent on ${\varvec{A}}$ regardless of the system’s behavior (reversible, quasi-reversible or irreversible). The ${\varvec{A}}$ of an electrode is also correlated with the current density $j$ (A cm^2^) observed during an electrochemical experiment, and thus its knowledge is necessary for obtaining the full picture of the electrochemical process under investigation. It is also involved in most calculations of other electrochemical parameters (${k}^{0}$, ${C}_{dl}$, etc.) and properties (for example, the electrocatalytic performance of a material) since most of them are often (and should be) normalized with ${\varvec{A}}$ [1–4]. Setting a clear pathway for its calculation is of paramount importance as it is probably one of the first studies an electrochemist should embark upon when evaluating a new system. Finally, as regards the screen-printed electrodes (SPEs) on which the case study is focused, it should be noted, that even though the inter-electrode reproducibility of a single batch of SPEs is rather satisfactory, it is highly recommended that the calculation of $A$ is performed at every new batch, as the status of both the ink and of the printing mesh as well as the printing settings can influence its value. This also applies for other types of non-conventional electrodes, such as the 3D-printed and the laser scribed electrodes, the $A$ of which is greatly affected by the fabrication settings, the source material etc.

There are two main techniques for the calculation of the electroactive area: i) chronocoulometry and ii) cyclic voltammetry, which will be presented in detail.

### Chronocoulometry

Chronocoulometry is a simple and commonly employed technique for the calculation of the electroactive area of an electrode, by performing experiments in the presence of a redox compound of known diffusion coefficient ($D$). As its name implies, chronocoulometry is the measurement of charge ($Q$, in coulombs) with time (chrono) and is implemented by applying a short *dc* potential step to an electrochemical cell, while the current (I, in amperes) transient is monitored. Charge *versus* time plots are provided by the electrochemical analyzers by integrating the current over time. In double potential step chronocoulometry, the potential of the working electrode (in fact, the potential difference *versus* the reference electrode) is stepped from an initial value (${E}_{i}$) where no electrochemical Faradaic reaction occurs to a value (${E}_{s}$) where complete electrolysis of the redox species occurs, so that the current is limited by (planar) diffusion, and then to a final value (${E}_{f}$), that is frequently identical to ${E}_{i}$ [5–7]. Obviously, experiments are conducted under quiescent conditions. The transition from ${E}_{i}$ to ${E}_{s}$ is commonly referred to as forward step, and from ${E}_{s}$ to ${E}_{f}(={E}_{i}$), as reverse step. The duration of each potential step is taken as $\tau$, in seconds.

The Anson equation (Eq. 1) calculates the total charge $$({Q}_{T})$$, which passes through the electrochemical cell at any time, due to the charging of the electrical double layer $$({Q}_{dl})$$ and the Faradaic reaction of diffusing ($${Q}_{diff})$$ and/or absorbed ($${Q}_{ads})$$ species of the redox compound, and can be applied to the calculation of the electroactive area of the working electrode $$({\varvec{A}})$$ [6, 8, 9].

Redox species are classified as outer- or inner-sphere depending on the way the electron transfer processes occur on the electrode’s surface [10]. In the case of outer-sphere redox species, the electrochemical process is diffusion controlled and thus is influenced by the electronic properties of the electrode surface only, while in the case of inner-sphere redox species, the electrochemical process is also influenced by surface functional groups and commonly involves adsorbed species [10–12].

In this regard, the Anson equation for a potential step when both diffusing and absorbed species are included in the electrochemical process is given by the following equation

1 $${Q}_{T}={Q}_{dl}+{Q}_{ads}+{Q}_{diff}={Q}_{dl}+{Q}_{ads}+\frac{2nFAC\sqrt{DT}}{\sqrt{\pi }}$$

where ${Q}_{T}$ is the total charge (in coulombs, $C$), $n$ is the number of electrons involved in the electrochemical reaction, $F$ is Faraday’s constant (96485 C moL^−1^), $A$ is the electrode’s electroactive area in cm^2^, $C$ is the concentration of the redox species in solution (moL cm^−3^), $D$ is the diffusion coefficient (cm^2^ s^−1^), while ${Q}_{dl}$, ${Q}_{ads}$ and ${Q}_{diff}$ are the charge components due to charging of the electrical double layer, the electrolysis of adsorbed species, and the electrolysis of solution (diffusing) species, respectively. The charge component ${Q}_{ads}$, if any, is equal to $nFA{\Gamma }_{0}$, where ${\Gamma }_{0}$ is the amount of adsorbed species on the electrode surface.

To discriminate among the three components of the total charge, ${Q}_{dl}, {Q}_{ads}$ and ${Q}_{diff}$, a plot of ${Q}_{T}$ *versus* $\sqrt{t}$ (during the forward step) and ($\sqrt{\tau }+\sqrt{t-\tau }-\sqrt{t})$ (during the reverse step), collectively termed as Anson plot, can be employed. Considering that ${Q}_{dl}$ and ${Q}_{ads}$ remain constant with the time, by using the Anson plot’s slope attributed to the diffusion-controlled charge transfer process (for an ideally reversible process the slopes at both forward and reverse steps should be the same), the electroactive area of the electrode can be calculated as per the following equation

2 $$slope \left(S\right)=\frac{2nFAC\sqrt{D}}{\sqrt{\pi }}$$

### Cyclic voltammetry

Considering the factors that should be taken into account regarding the size and the morphology of the electrodes (for a detailed analysis of these factors the reader is referred to the Refs [11, 13–15]) if cyclic voltammetry is employed, the electrode electroactive area can be calculated, in the presence of a redox species in the measuring solution, by using the proper variation of the Randles-Ševčík equation. In the case of a reversible process (${n\Delta E}_{p}\approx 57.5$ mV, ${\Delta E}_{p}$ is the peak-to-peak potential separation), the peak current is described by the Randles-Ševčík equation

3 $${I}_{p}=2.69\times {10}^{5}{n}^{3/2}A\sqrt{D}C\sqrt{v}$$

where ${I}_{p}$ is the forward peak current, $v$ is the potential scan rate, while the other terms have the aforesaid meaning. Thus, for a reversible system, the peak current is proportional to the square root of the scan rate. A plot of ${I}_{p}$ *versus* $\sqrt{v}$ gives a straight line, the slope of which can be used to calculate the ${\varvec{A}}$ [2, 5, 9].

**Fig. 1:**
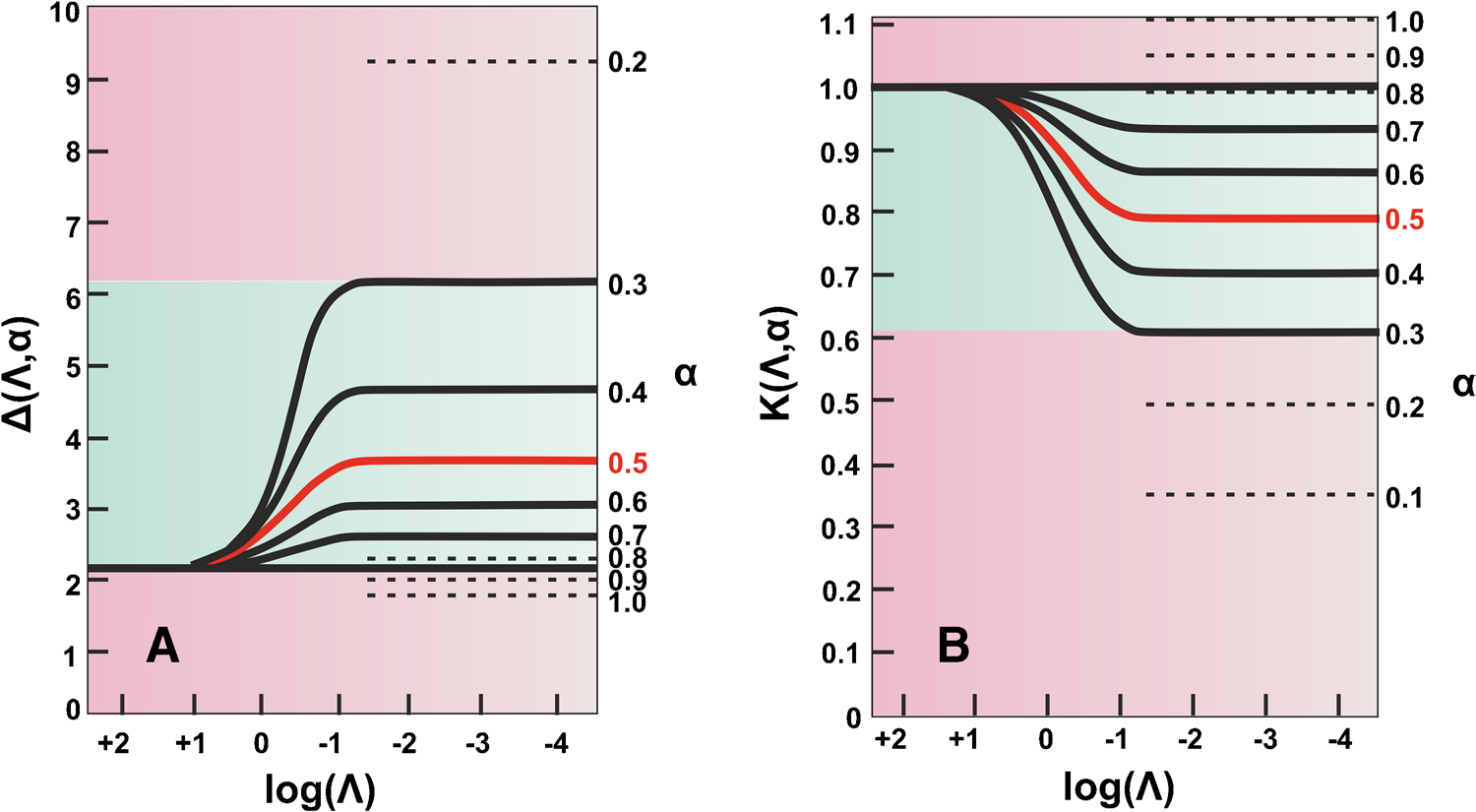
Variation of (**A**) $\Delta (\Lambda ,\alpha )$ and (**B**) ${\rm K}(\Lambda ,\alpha )$ with $\Lambda$ for different values of $\alpha$. Adapted with permission from [2], Copyright (2001) Wiley

***Note***: We will see below (eqs. 11 and 12) that the $\Psi$ parameter is given as a function of ${\Delta E}_{p}$, which in turn, for a non-reversible process is dependent on the scan rate. Since $K\left(\Lambda ,\alpha \right)=f\left(\mathrm{log}\Lambda \right)=f\left(\Psi \right)=f(\sqrt{v})$, a different $K\left(\Lambda ,\alpha \right)$ value emerges for each different scan rate value [2]. In other words, the $K\left(\Lambda ,\alpha \right)$ parameter in Eq. 4, declares that at a quasi-reversible process ($63<{n\Delta E}_{p}<200$ mV), the peak current is not proportional to the square root of scan rate.

Nevertheless, despite the popular bibliographical belief, common redox molecules (for example, potassium ferrocyanide) are more often involved in quasi-reversible processes (typically, $63<{n\Delta E}_{p}<200$ mV), especially when non-conventional electrode materials, such as the widely used in modern electro-sensing applications, screen-printed or 3D-printed electrodes, are employed. In these cases, if $63<{n\Delta E}_{p}<200$ mV, Eq. 3 is not valid and the modified Randles–Ševčík equation for quasi-reversible processes should be used instead [16]

4 $${I}_{p}=(2.69\times {10}^{5}{n}^{3/2}A\sqrt{D}C\sqrt{v}) {\rm K}(\Lambda ,\alpha )$$

where ${\rm K}(\Lambda ,\alpha )$ is a modified dimensionless parameter for quasi-reversible reactions. To estimate the $\boldsymbol{\rm K}(\boldsymbol{\Lambda },\boldsymbol{\alpha })$ parameter, we have first to calculate another dimensionless parameter termed $\boldsymbol{\Delta }(\boldsymbol{\Lambda },\boldsymbol{\alpha })$ by using Eq. 5, and parameter $\boldsymbol{\Lambda }$ by using Eq. 6 [17]

5 $${E}_{p/2}-{E}_{p}=\Delta \left(\Lambda , \alpha \right)\left(\frac{RT}{F}\right)=26 \Delta \left(\Lambda , \alpha \right) \left(\mathrm{at }25^\circ \mathrm{C}\right)$$

where ${E}_{p}$ is the peak potential, ${E}_{p/2}$ is the half-wave potential (these values can be found from the recorded cyclic voltammograms), $R$ is the gas constant (8.314 J mol^−1^ K^−1^) and $T$ is the temperature ($K$).

6 $$\Lambda =\Psi \sqrt{\pi }$$

where $\Psi$ is a kinetic parameter and its calculation is described analytically below. The next step is a simple calculation of $\mathrm{log}\Lambda$. Using the plot $\Delta \left(\Lambda ,\alpha \right)=f(\mathrm{log}\Lambda )$ (Fig. 1A), as the parameters $\Delta (\Lambda ,\alpha )$ and $\mathrm{log}\Lambda$ are known, the transfer coefficient ($\alpha$) can be approximated graphically. Finally, the parameter ${\rm K}(\Lambda ,\alpha )$ is found graphically from the plot $K\left(\Lambda ,\alpha \right)=f(\mathrm{log}\Lambda )$ for the estimated value of $\alpha$ (Fig. 1B) [2, 18].

In the case of an irreversible process (typically, when $n{\Delta E}_{p}>200$ mV), the peak current is given by the following equation

7 $${I}_{p}=2.99 \times {10}^{5}n\sqrt{{\alpha n}^{\mathrm{^{\prime}}}}A\sqrt{DC}\sqrt{v}$$

where $n$ and ${n}^{^{\prime}}$ are the number of electrons in the electrochemical reaction and the number of electrons transferred before the rate determining step, respectively [11, 19], while all the other terms have their aforementioned meaning. Assuming that $n={n}^{^{\prime}}=1$, the peak current is calculated by using the modified Randles-Ševčík equation given below

8 $${I}_{p}=2.99\times {10}^{5}\sqrt{\alpha }A\sqrt{D}C\sqrt{v}$$

The transfer coefficient ($\alpha$) is a measure of symmetry of the energy barrier for a single electron transfer step [2]. For a purely symmetrical reaction in terms of energy (a reversible reaction) has a transfer coefficient of 0.5 [1, 19]. Consequently, in irreversible reactions $\alpha$ should not be considered to be 0.5. In this case, $\alpha$ can be calculated by the Eq. 9 [2]

9 $$\left|{E}_{p}-{E}_{p/2}\right|=\frac{1.857RT}{\alpha F}=\frac{47.7}{\alpha } \left(\mathrm{at }25 ^\circ \mathrm{C}\right)$$

### *Calculation of heterogeneous electron transfer rate constants (*${k}^{0}$*)*

Heterogeneous electron transfer rate constants (${k}^{0}$) have been a key parameter of the electrochemical performance of various electrode materials, or electrode modifications, or electrode modifiers since they reflect the kinetics of the reaction between a particular redox compound and the electrode surface [19]. In general, ${k}^{0}$ is a measure of the heterogeneous kinetic facility between the oxidized and the reduced forms of a redox couple. An electrochemical system with a large ${k}^{0}$ can achieve equilibrium quickly, while a system with small ${k}^{0}$, sluggishly [2]. Admittedly, cyclic voltammetry is by far the most employed technique for the calculation of ${k}^{0}$, since several methods (Nicholson [20], Klingler-Kochi [21], Gileadi [22], etc.) have been developed on it. Besides, even though not so widely used, electrochemical impedance spectroscopy (EIS) can also be used for the calculation of ${k}^{0}$ both in a numerical [16, 23–25] and in a graphical fashion [26].

***Note:*** The rate constant of an electrode reaction refers to the time needed for the electroactive species to arrange themselves and their ionic atmospheres for the electron transfer to occur. It does not measure the rate of electron transfer itself, as this occurs extremely rapidly, in approximately 10^−16^ s [1].

### Cyclic voltammetry-based methods

#### ***Nicholson method ***[20]

One of the most commonly used voltammetry-based methods is Nicholson’s method [20], in which ${k}^{0}$ for quasi-reversible electrochemical reactions is related to a dimensionless kinetic parameter, named $\Psi$, as per Eq. 10

10 $${k}^{o}=\Psi \sqrt{\frac{\pi Dn\nu F}{RT}}$$

where $\Psi$ works as a polynomial function of the product $n{\Delta E}_{p}$ within the range $63<n{\Delta E}_{p}<212$ mV, or with respect to $\Psi$ values, within the ($7>\Psi >0.1$) range. The upper $\Psi$ limit of 7 (or $n{\Delta E}_{p}>63$ mV) signifies the transition between a reversible to a quasi-reversible system, while the lower limit of 0.1 (or $n{\Delta E}_{p}>212$ mV) signifies the transition between a quasi-reversible system to an irreversible one. Practically, the only input required to calculate ${k}^{0}$ is the value of the $\Psi$ parameter, provided that the $n{\Delta E}_{p}$ value is within the quasi-reversible limits ($63<n{\Delta E}_{p}<212$ mV) set above. Obviously, the ${n\Delta E}_{p}$ value is highly dependent on the experimental variables (the concentration of the redox compound, the electrolyte, the pH of the measuring solution, the scan rate, etc.), which can thus be appropriately tuned to set ${n\Delta E}_{p}$ within the quasi-reversible limits ($63<n{\Delta E}_{p}<212$ mV), where the Nicholson method is valid.

**Fig. 2:**
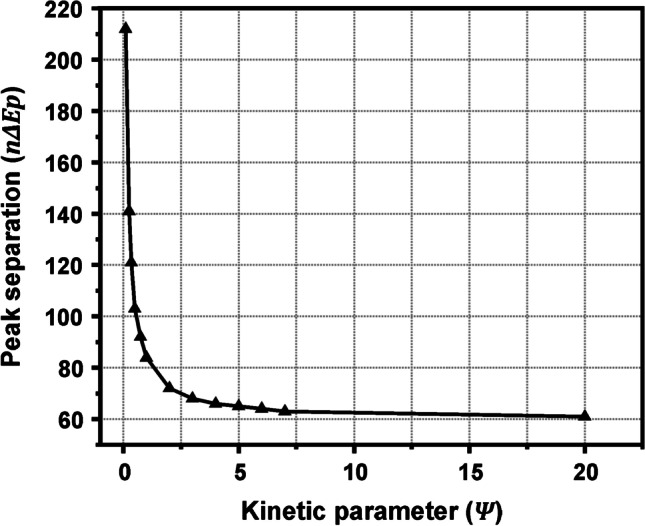
Working curve showing variation of ${n\Delta E}_{p}$ with $\Psi$. The plot was constructed by using the values given in Table 1 in Ref. [20]

The $\Psi$ parameter can be found graphically from Fig. 2 (this graph represents a better resolution graph of Fig. 3 in [20] and it was constructed based on the data given in Table 1 of [20]). More conveniently, $\Psi$ parameter can be calculated by using the empirical equation

11 $$\Psi =\frac{-0.6288+0.0021(n{\Delta E}_{p})}{1-0.017(n{\Delta E}_{p})}$$

developed by Lavagnini et al. [27]. In fact, Eq. 11 is a polynomial fit of Nicholson’s working curve and allows the easy and accurate determination of $\Psi$ parameter from the ${n\Delta E}_{p}$ of a single cyclic voltammogram. When $n{\Delta E}_{p}$ ranges between 140 and 200 mV, the same authors [27] suggest that $\Psi$ parameter to be calculated by using the following equation

12 $$\Psi =2.18 \sqrt{\frac{\alpha }{\pi }}{ e}^{-(\frac{{a}^{2}nF}{RT}){\Delta E}_{p}}$$

which offers a more representative $\Psi$ value in this range.

**Fig. 3:**
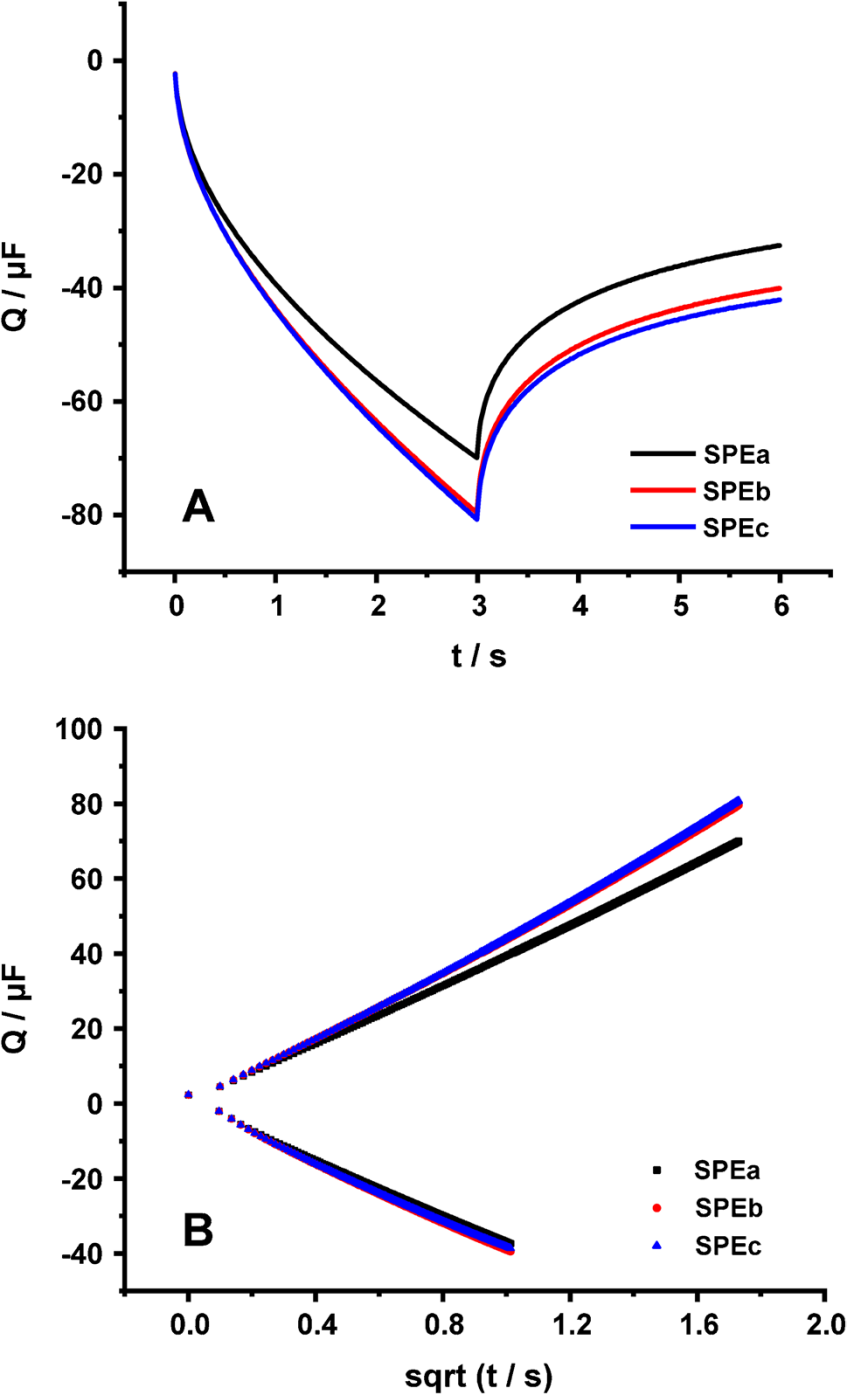
**(A)** Chronocoulograms and **(B)** the respective Anson plots of three different graphite SPEs in 0.1 M KCl containing 1 mM RuHex (charge response in 0.1 M KCl has been subtracted)

**Table 1:** An overview of commonly used techniques and methods for the calculation of $A$, and ${k}^{o}$

Technique/method	Equations	Comments
Electroactive area (A)		
Chronocoulometry	$Slope=2nFAC\sqrt{D}/\sqrt{\pi }$	Transformation of chronocoulometry data to Anson plots and calculation of $A$ by the respective slopes
Cyclic voltammetry	Reversible process: ${I}_{p}=2.69\times {10}^{5}{n}^{3/2}A\sqrt{D}C\sqrt{v}$	${n\Delta E}_{p}=59 mV ($ in practice lower than 63 mV) The calculation of $A$ from the slope of ${I}_{p}=f(\sqrt{v})$ and not by applying the equation to a single scan rate is suggested
	Quasi-reversible process: ${I}_{p}=(2.69\times {10}^{5}{n}^{3/2}A\sqrt{D}C\sqrt{v}) {\rm K}(\Lambda ,\alpha )$	$63<{n\Delta E}_{p}<200$ mV ${\rm K}\left(\Lambda ,\alpha \right)$ is found as follows: i) calculation of $\Psi$ (see below in this column), ii) calculation of $\mathrm{log}\Lambda$ as $\Lambda =\Psi \sqrt{\pi }$, iii) calculation of $\Delta \left(\Lambda , \alpha \right)$ as $\Delta \left(\Lambda , \alpha \right)=\left({E}_{p/2}-{E}_{p}\right)/26$, iv) $a$ can be found graphically from the plot $\Delta \left(\Lambda , \alpha \right)=f(\mathrm{log}\Lambda )$, v) ${\rm K}\left(\Lambda ,\alpha \right)$ can be found graphically from the plot ${\rm K}\left(\Lambda ,\alpha \right)=f(\mathrm{log}\Lambda )$ for the $a$ value approximated in step iv
	Irreversible process: ${I}_{p}=2.99\times {10}^{5}{\alpha }^{1/2}A\sqrt{D}C\sqrt{v}$	${n\Delta E}_{p}>200$ mV α can be calculated by $\left|{E}_{p}-{E}_{p/2}\right|=47.7/\alpha$
Heterogeneous electron transfer rate (k^o^)		
Cyclic voltammetry/Nicholson method	${k}^{o}=\Psi \sqrt{\pi DnvF/RT}$	$\Psi$ can be found graphically from Nicholson plot [20] ($n\Delta Ep$ up to 212 mV; see Fig. 2) or can be calculated from the respective fitting: $\Psi =\left(-0.6288+0.0021{\Delta E}_{p}\right)/\left(1-0.017{\Delta E}_{p}\right)$ ($n\Delta Ep$ up to 200 mV) [27] or $\Psi =2.18 \sqrt{\alpha /\pi }{ e}^{-({a}^{2}nF/RT){\Delta E}_{p}}$ (150 < $n\Delta Ep$ < 200 mV) [27]
Cyclic voltammetry/Klingler-Kochi method	${k}^{o}=2.18\sqrt{\alpha DnvF/RT}{e}^{-{\alpha }^{2}nF{\Delta E}_{p}/RT}$	$n\Delta Ep$> 150 mV α can be calculated by $\left|{E}_{p}-{E}_{p/2}\right|=47.7/\alpha$
Cyclic voltammetry/Gileadi method	$\mathit{log}{k}^{o}= -0.48\alpha +0.52+log\sqrt{nF\alpha {v}_{c}D/2.303RT}$	${v}_{c}$ is estimated graphically through the ${E}_{p}=f (\mathrm{log}v)$ plot
Electrochemical Impedance Spectroscopy	${k}^{o}=RT/{n}^{2}{F}^{2}{R}_{ct}AC$	On condition that impedance spectrum fitting Randles circuit and that the overpotential of the redox probe is low (typically < 50 mV; see Fig. 9) ${R}_{ct}$ is calculated by fitting of the Nyquist plot

***Note:*** An alternative graphical approach is plotting the Eq. 10 as an $f(x)=bx$ function, where $f(x)=\Psi$, $x=1/\sqrt{\pi Dn\nu F/RT}$ and $b={k}^{0}$ (slope) at different scan rate values, with the restriction of $n\Delta {E}_{p}$ being kept under 212 mV. As a general rule, the average value of ${k}^{o}$ at different scan rates is considered more reliable than its value at a single scan rate.

#### ***Klingler-Kochi method ***[21]

In the case of irreversible systems ($n{\Delta E}_{p}>150$ mV), the following equation suggested by Klingler and Kochi [21] is available and should be considered:

13 $${k}^{0}=2.18\sqrt{\frac{\alpha Dn\nu F}{RT}}{ e}^{-(\frac{{a}^{2}nF}{RT}){\Delta E}_{p}}$$

where $\alpha$, the transfer coefficient of the forward scan, is calculated as described above (Eq. 9) and all the other symbols have the same meaning.

***Note:*** when ${n\Delta E}_{p}>150$ mV, by using the Klingler and Kochi method [21] and Eq. 13, ${k}^{0}$ can be directly calculated from a single experimental variable (${n\Delta E}_{p}$) without being necessary any other parameter (for example, $\Psi$) to be previously calculated. Remember though that the average value at different scan rates is preferred.

#### ***Gileadi method ***[22]

In this method, there is no restriction to the ${\Delta E}_{p}$ value since it is based on the graphical approximation of the critical scan rate (at which the electrochemical process changes from reversible or quasi-reversible to irreversible) from a plot presenting the variation of the peak potential with the logarithm of scan rate [${E}_{p}=f(\mathrm{log}v)$]. Two straight lines with different slopes are obtained at the low and high scan rates and the value of the critical scan rate is estimated from the intersection of the extrapolated lines. Then, the calculation of ${k}^{0}$ is performed using the following equation

14 $$\mathit{log}{k}^{o}= -0.48\alpha +0.52+log\sqrt{\frac{nF\alpha {v}_{c}D}{2.303RT}}$$

where ${v}_{c}$ is the critical scan rate and the other terms have their aforesaid meaning [22, 28].

***Note:*** The methods presented above, and especially the Gileadi method which is based on the graphical calculation of ${k}^{0}$ should not be confused with the well-known method of Laviron [29], which refers to surface confined redox species and as a result the respective rate constant is given in s^−1^ and not in cm s^−1^.

#### EIS-based methods

EIS enables the calculation of ${k}^{0}$ through the determination of charge transfer resistance (${R}_{ct}$) when EIS measurements are conducted in the presence of a redox couple (the electrolyte contains both the Ox and Red forms of a redox system). The relationship between ${k}^{0}$ and EIS derived data has been introduced by Randles [30], and has been studied extensively by Sluyters [23]. The apparent limitation of this method is that the impedance spectrum must be sufficiently modeled by a Randles equivalent electrical circuit [26] and that the overpotential of the redox reaction should be sufficiently low (see Results & Discussion).

***Note:*** ${k}^{0}$ may be also determined by EIS when examining complex electrochemical that cannot be simulated to a single Randles circuit. In those cases, profound knowledge of the electrochemical system at hand is essential because the existence of additional charge-transfer phenomena can lead to the existence of more than one ${R}_{\mathrm{ct}}$ values in the equivalent circuit.

The estimation of ${k}^{0}$ by EIS data is possible when working at the linear part of the Butler-Volmer equation, valid for small overpotential values [2]. Under these conditions, the exchange current ${i}_{0}$ is related with the (small) values of ${R}_{ct}$ with the Eq. 15

15 $${i}_{0}= \frac{RT}{nF{R}_{ct}}$$

Exchange current ${i}_{0}$ is also correlated with ${k}^{0}$ according to Eq. 16:

16 $${i}_{0}=nFA{k}^{0}{C}_{ox}^{*\alpha }{C}_{red}^{*1-\alpha }$$

where ${C}^{*}={C}_{ox}^{*}={C}_{red}^{*}$_,_ is the concentration of the redox couple in the solution. By combining eqs. 15 and 16, the correlation between ${R}_{ct}$ and ${k}^{0}$ is enabled as follows:

17 $${k}^{0 }= \frac{RT}{{n}^{2}{F}^{2}AC{R}_{ct}}$$

***Note:*** An alternative graphical estimation of ${k}^{0}$ can be used if we inverse Eq. 17 and consider it as an $f(x)=bx$ function, where $f(x)={R}_{ct}$, $x=1/C$ and $b=slope=RT/{n}^{2}{F}^{2}A{k}^{0}$, at increasing concentrations of the redox probe, which will result at decreasing ${R}_{ct}$ [26].

**Note**: For an ideally reversible reaction the overpotential is zero and consequently so is the exchange current. By extension, that means that the ${R}_{ct}$ is also zero and ${k}^{0}$ tends to infinity (${R}_{ct}$=0, ${k}^{0}\to \infty$) [8].

An overview of the various techniques and methods elaborated in this case study is summarized in Table 1, while the experiments conducted, and the respective results are presented in Table 2.

**Table 2:** An overview of the techniques and methods used in this case study, as well as the obtained results. Values are presented as mean ± SD, n = 3. CC, chronocoulometry; CV, cyclic voltammetry; EIS, electrochemical impedance spectroscopy

Technique	Redox probe	Electrolyte	Calculated parameter	Electroactive Area^*^(A / cm^2^)	Heterogeneous electron transfer rate (10^−3^) k^o^ / cm s^−1^
CC	1 mM RuHex	0.1 M KCl	$A$	0.1154 ± 0.0032	−
CC	1 mM ferricyanide	0.1 M KCl, pH 3	$A$	0.1510 ± 0.0052	−
CC	1 mM ferricyanide	0.1 M PBS, pH 7	$A$	0.1481 ± 0.0087	−
CV	1 mM RuHex	0.1 M KCl	$A, {k}^{o}$	0.1002 ± 0.0023^[a]^	2.535 ± 0.039^[g]^
					2.495 ± 0.078^[h]^
				0.1015 ± 0.0010^[b]^	
					3.509 ± 0.143^[i]^
CV	1 mM ferricyanide	0.1 M KCl, pH 3	$A, {k}^{o}$	0.0990 ± 0.0023^[c]^	1.182 ± 0.150^[j]^
				0.0941 ± 0.0058^[d]^	2.197 ± 0.128^[k]^
				0.0951 ± 0.0021^[e]^	3.310 ± 0.250^[l]^
CV	1 mM ferricyanide	0.1 M PBS, pH 7	$A, {k}^{o}$	0.0331 ± 0.0035^[f]^	0.401 ± 0.022^[m]^
EIS	1 + 1 mM ferro/ferricyanide	0.1 M PBS, pH 7	${k}^{o}$	-	0.022 ± 0.002

^[a]^calculated by the Eq. 4 at $v$ = 5 – 300 mV s^−1^ ($\Delta {E}_{p}$ = 86 – 192 mV)

^[b]^calculated from the slope of ${I}_{p}/L=f(\sqrt{v})$ plot in which ${I}_{p}$ have been corrected with the $L$ parameter corresponding either to the $K\left(\Lambda ,\alpha \right)$ (at $v$ = 5 – 300 mV s^−1^, $\Delta {E}_{p}$ = 86 – 192 mV) or to $1.11\sqrt{\alpha }$ (at $v$ = 350 – 500 mV s^−1^, $\Delta {E}_{p}$ = 203 – 223 mV)

^[c]^calculated by the Eq. 4 at $v$ = 5 – 50 mV s^−1^ ($\Delta {E}_{p}$ = 113 – 198 mV)

^[d]^calculated by the Eq. 8 at $v$ = 75 – 500 mV s^−1^ ($\Delta {E}_{p}$ = 227 – 394 mV)

^[e]^calculated from the slope of ${I}_{p}/L=f(\sqrt{v})$ plot in which ${I}_{p}$ have been corrected with the $L$ parameter corresponding either to the $K\left(\Lambda ,\alpha \right)$ (at $v$ = 5 – 50 mV s^−1^, $\Delta {E}_{p}$ = 113 – 198 mV) or to $1.11\sqrt{\alpha }$ (at $v$ = 75 – 500 mV s^−1^, $\Delta {E}_{p}$ = 227 – 394 mV)

^[f]^calculated by the Eq. 8 at $v$ = 5 – 500 mV s^−1^ ($\Delta {E}_{p}$ = 322 – 864 mV)

^[g]^calculated by the Nicholson method at $v$ = 5 – 300 mV s^−1^ ($\Delta {E}_{p}$ = 86 – 192 mV)

^[h]^calculated by the Klingler-Kochi method at $v$ = 150 – 500 mV s^−1^ ($\Delta {E}_{p}$ = 159 – 223 mV)

^[i]^calculated by the Gileadi method

^[j]^calculated by the Nicholson method at $v$ = 5 – 50 mV s^−1^ ($\Delta {E}_{p}$ = 113 – 198 mV)

^[k]^calculated by the Klingler-Kochi method at $v$ = 25 – 500 mV s^−1^ ($\Delta {E}_{p}$ = 164 – 394 mV)

^[l]^calculated by the Gileadi method

^[m]^calculated by the Klingler-Kochi method at $v$ = 5 – 500 mV s^−1^ ($\Delta {E}_{p}$ = 322 – 864 mV)

## Experimental

### Materials

Hexaammineruthenium(III) chloride (RuHex) was purchased from Aldrich. Potassium hexacyanoferrate(III) (ferricyanide) was purchased by AnalaR. Potassium hexacyanoferrate(II) trihydrate (ferrocyanide) was a product of Merck. All the other reagents were of analytical grade from Sigma-Aldrich.

### Apparatus

Electrochemical measurements were conducted with a PGSTAT12/FRAII electrochemical analyzer (Metrohm Autolab) in a three-electrode cell. A Pt wire and a Ag/AgCl/3 M KCl electrode (IJ Cambria) were used as the counter electrode and the reference electrode, respectively, while graphite SPEs served as the working electrodes. Chronocoulometry experiments were conducted in a) 0.1 M KCl in the absence and presence of 1 mM RuHex, b) 0.1 M KCl, pH 3 in the absence and presence of 1 mM ferricyanide, and c) 0.1 M phosphate buffered saline (PBS, pH 7) in the absence and presence of 1 mM ferricyanide. Cyclic voltammograms were recorded in a) 0.1 M KCl containing 1 mM RuHex, b) 0.1 M KCl, pH 3 containing 1 mM ferricyanide, c) 0.1 M PBS, pH 7 containing 1 mM ferricyanide, and d) 0.1 M KCl, pH 3 or PBS, pH 7 containing 1 + 1 mM ferro/ferricyanide at different scan rates. EIS spectra were recorded in a mixture of ferro/ferricyanide in 0.1 M KCl, pH 3 or 0.1 M PBS, pH 7 over the frequency range from 100 kHz to 0.1 Hz by using a sinusoidal excitation signal of 0.010 V (rms) superimposed on a *dc* potential. The applied *dc* potential was 0.25 and 0.21 V for KCl and PBS, respectively. The results in the text refer to the mean value and the standard deviation of measurements with three different SPEs.

### Fabrication of graphite SPEs

The graphite SPEs (4 mm diameter) were fabricated in arrays of twenty electrodes onto a 175 μm polyester substrate (Autostat CUS7) using a semi-automatic screen printer (E2, EKRA) and home-made polyester screens with 195 mesh (77/195–48 PW, SEFAR PET 1500). Graphite ink (Loctite EDAG 407A) was pushed into the open area of the screens with a 75-durometer polyurethane squeegee. SPEs were cured at 90 °C for 60 min in a conventional oven.

## Results and discussion

### *Calculation of electroactive area (*$A$*)*

Chronocoulometric and cyclic voltammetry experiments using two different redox probes (an outer-sphere, RuHex, and an inner-sphere, ferricyanide) were conducted, and the electrode electroactive area was calculated with the various techniques/methods discussed above. Calculated values are also expressed as the percentage ratio of the calculated area ($A$) to the geometric area (${A}_{geo}$) ($\%R=A/{A}_{geo}\times 100$) [11]. The ${A}_{geo}$ of SPEs (d = 0.4 cm) is 0.1256 cm^2^.

As regards the electroactive area calculated using double potential step chronocoulometry, measurements were conducted in 1 mM RuHex in 0.1 M KCl or 1 mM ferricyanide in 0.1 M KCl, pH 3 or 0.1 M PBS, pH 7. The values of ${E}_{s}$ and ${E}_{i}={E}_{f}$ for RuHex were − 0.5 V and 0.2 V and for ferricyanide − 0.4 V and 0.8 V, respectively. The total charge that passed through the cell was recorded for 6 s (3 s at ${E}_{s}$ and 3 s at ${E}_{f}$) to ensure that the redox probe will diffuse to the electrode at its maximum rate [2], in accordance with previous studies [11]. Chronocoulometric measurements (3 s at ${E}_{s}$ and 3 s at ${E}_{f}$) were preceded by a conditioning step at ${E}_{i}$ for 3 s. The experiments were conducted with 3 different SPEs. The signal recorded in the absence of the redox probe (in pure electrolyte) was subtracted from the signal recorded in the presence of the redox probe. The subtracted chronocoulometric signals of three different SPEs in 0.1 M KCl containing 1 mM RuHex and the respective Anson plots are presented in Fig. 3. Generally, while the calculation of $A$ is feasible using either slope of the Anson plot, we have selected the one that corresponds to the ${E}_{f}$ in each case, since it presents the most reproducible results, and the linearity of the respective curves is better. The diffusion coefficients used in this work were 9.1 × 10^−6^ and 7.6 × 10^−6^ cm^2^ s^−1^ for RuHex and ferricyanide, respectively. The electroactive area calculated using RuHex is 0.1154 ± 0.0032 cm^2^ ($\%R$ = 91.9%), while the areas calculated using ferricyanide in KCl, pH 3 and in PBS, pH 7 are 0.1510 ± 0.0052 ($\%R$ = 120.2%) and 0.1481 ± 0.0087 cm^2^ ($\%R$ = 117.9%), respectively.

**Fig. 4:**
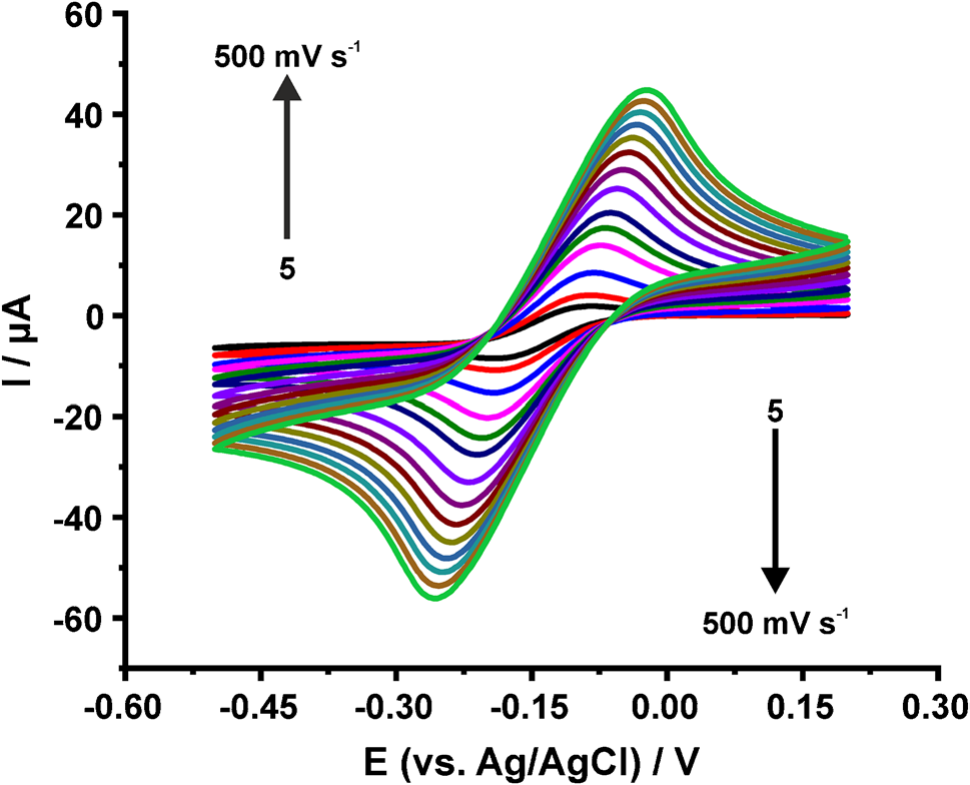
CVs of graphite SPE in 0.1 M KCl containing 1 mM RuHex at different scan rates

**Fig. 5:**
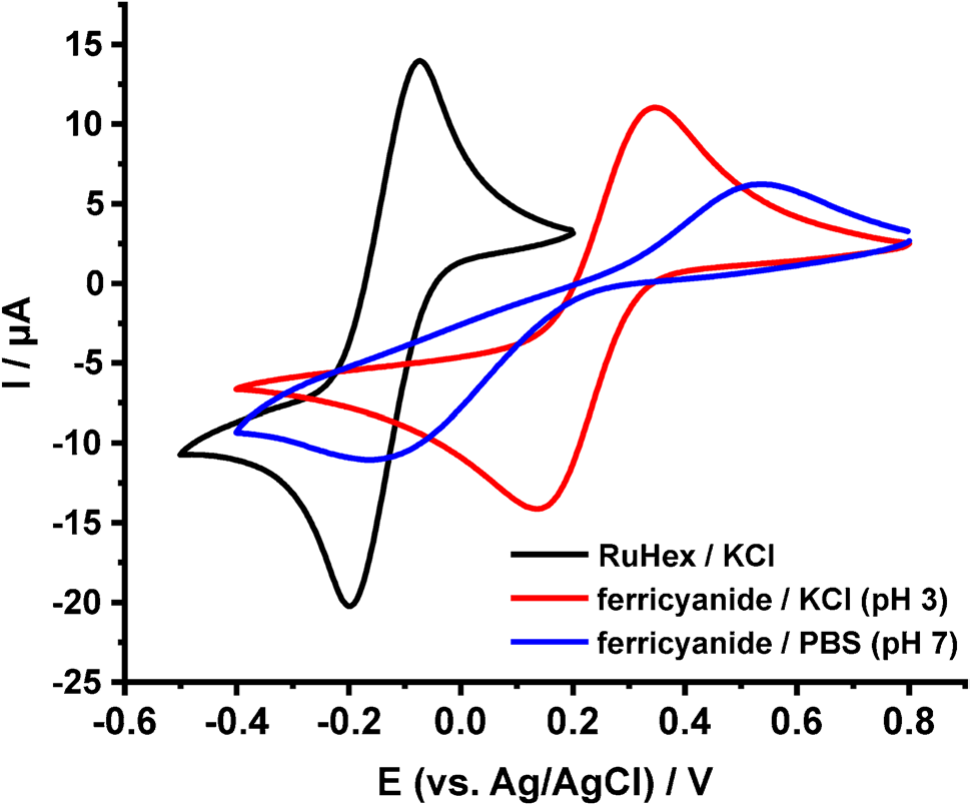
CVs of graphite SPEs in 0.1 M KCl containing 1 mM RuHex, 0.1 M KCl, pH 3 containing 1 mM ferricyanide, and 0.1 M PBS, pH 7 containing 1 mM ferricyanide. Scan rate: 50 mV s.^−1^

To calculate the electrochemically active area of the electrodes via cyclic voltammetry, by using, depending on the $\Delta {E}_{p}$ for each recorded CV, the appropriate in each case Randles-Ševčík equation, CVs in 1 mM RuHex in KCl, 1 mM ferricyanide in 0.1 M KCl, pH 3 and 1 mM ferricyanide in 0.1 M PBS, pH 7 at different scan rates (5, 10, 25, 50, 75, 100, 150, 200, 250, 300, 350, 400, 450, and 500 mV s^−1^) were recorded. Although there are many examples in literature showing that a CV measurement at a single scan rate can yield useful information, we suggest running a series of CV measurements at different scan rates. Then, $A$ can be averaged from the calculated values in each scan rate or can be found graphically from the appropriate ${I}_{p}=f\left(\sqrt{v}\right)$ plot. As an example, the CVs obtained with a graphite SPE in 0.1 M KCl containing 1 mM RuHex in 0.1 M KCl at different scan rates are depicted in Fig. 4, while to compare the CV responses at the three examined measuring solutions, the CVs obtained in each case at the same scan rate value (50 mV s^−1^) are presented in Fig. 5.

From the CVs in 0.1 M KCl containing 1 mM RuHex, the average electroactive area of 3 SPEs was calculated for scan rates 5 − 300 mV s^−1^, where $63<\Delta {E}_{p}<200$ mV, using the Randles-Ševčík equation for quasi-reversible processes (Eq. 4) and was found 0.1002 ± 0.0023 cm^2^ ($\%R$ = 79.8%). From the CVs in ferricyanide in 0.1 M KCl, pH 3, average $A$ was calculated from the scans conducted at scan rate 5 − 50 mV s^−1^, with the modified Randles-Ševčík equation for quasi-reversible processes (Eq. 4), while from the CVs conducted at scan rates 75 − 500 mV s^−1^, due to the large $\Delta {E}_{p}$ at the respective CVs, with the Randles-Ševčík equation for irreversible processes (Eq. 8). $A$ was calculated 0.0990 ± 0.0023 ($\%R$ = 78.8%) and 0.0941 ± 0.0058 cm^2^ ($\%R$ = 74.9%), respectively. From the measurements in ferricyanide in 0.1 M PBS pH 7, due to the large $\Delta {E}_{p}$ at all the examined scan rates, average $A$ was also calculated by using the Randles-Ševčík equation for irreversible processes (Eq. 8) and was found to be 0.0331 ± 0.0035 cm^2^ ($\%R$ = 26.3%). $a$ was calculated for each scan rate value at which the cyclic voltammetric response become irreversible (at high scan rates in ferricyanide in 0.1 M KCl, pH 3 or at all scan rates in ferricyanide in 0.1 M PBS, pH 7) using Eq. 9 and was used accordingly.

Concerning the values of $A$ calculated by chronocoulometric experiments with RuHex in KCl and with ferricyanide in 0.1 M KCl, pH 3 or 0.1 M PBS, pH 7, the former was found to be near to ${A}_{geo}$ ($\%R$ = 91.9%) in contrast with the two values obtained with ferricyanide, which are considerably higher ($\%R$ = 120.2 and 117.9%).

From the values of $A$ calculated by cyclic voltammetry measurements, the experiment conducted with ferricyanide in 0.1 M PBS, pH 7 seems to be a poor choice ($\%R$ was only 26.3%), while the results obtained from the other experiments could be considered satisfying. Data indicate that the modified Randles-Ševčík equation for irreversible systems cannot provide safe results for the calculation of $A$, especially when the $\Delta {E}_{p}$ values are too high (500 to 800 mV). This is not the case, however, for the ferricyanide in 0.1 M KCl, pH 3, especially at low scan rates (5–50 mV s^−1^) where the respective $\Delta {E}_{p}$ values indicate a quasi-reversible response.

To summarize, the electroactive area calculated with RuHex via chronocoulometry, and the respective values calculated with the same redox probe via cyclic voltammetry, as well as with ferricyanide in 0.1 M KCl, pH 3 at low scan rates are quite similar. Even though we cannot compare the obtained results with respect to a reference value, chronocoulometric measurements using RuHex seems to be more advantageous than the others for the calculation of the electroactive area of graphite SPEs, since it offers fast and reliable results (considering the calculated $\%R$ in each case), in agreement with previous works [11].

**Fig. 6:**
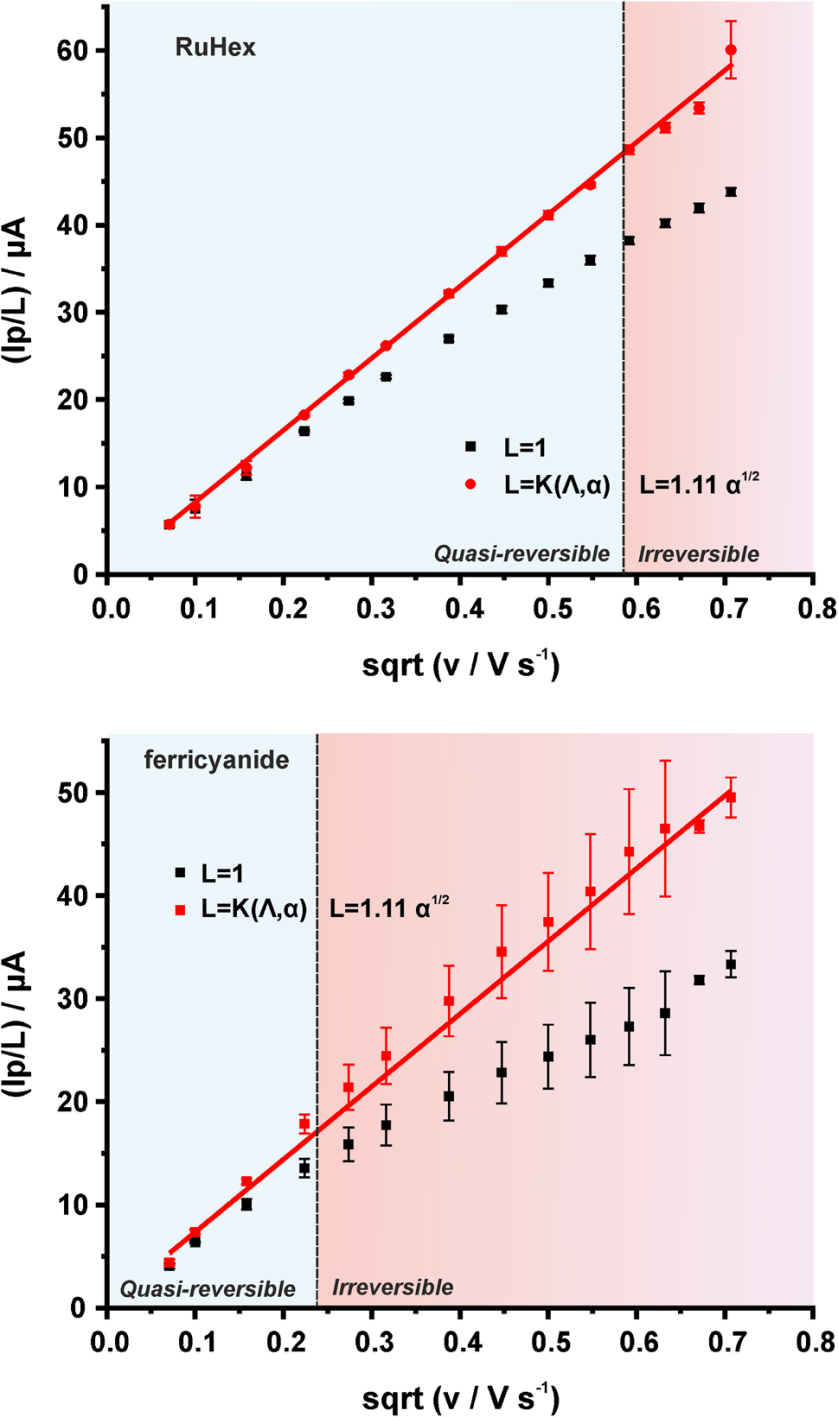
Plot of ${I}_{p}/L$ obtained from the CVs of graphite SPEs in (**A**) 0.1 M KCl containing 1 mM RuHex and (**B**) 0.1 M KCl, pH 3 containing 1 mM ferricyanide against the square root of scan rate

Quite often in the literature, in the case of quasi-reversible processes the calculation of $A$ has been conducted incorrectly using the Randles-Ševčík equation for reversible processes, and not by using the modified Randles-Ševčík equations. This predication that the ${I}_{p}$ is proportional to the $\sqrt{v}$ in any case leads to an important error. Additionally, in irreversible reactions a similar error occurs when $\alpha$ is considered 0.5, and not calculated by the ${E}_{p}$ and ${E}_{p/2}$ values (see Eq. 9) at each examined scan rate. To clear these resulting errors up, we demonstrate the use of a ${I}_{p}/L=f\left(\sqrt{v}\right)$ function, where “$L$” (which in this case $L$ stands for linearity) is the necessary divisor for maintaining the linear dependence of ${I}_{p}$ to the $\sqrt{v}$ (Fig. 6). More specifically, in the case of quasi-reversible processes the $L$ factor is equal to the $K(\Lambda , \alpha )$ (see Eq. 4) and in the case of irreversible processes the factor $L$ is equal to $1.11\sqrt{\alpha }$ (see Eq. 8). The discrimination of the electrochemical process as “quasi-reversible” or “irreversible” is based on the value of $n\Delta {E}_{p}$ at each CV. When $n\Delta {E}_{p}<200 mV$, the ${I}_{p}$ value is corrected to the corresponding $K(\Lambda , \alpha )$ value as dictated by Eq. 4 for a quasi-reversible process, and when $n\Delta {E}_{p}>200 mV$, the ${I}_{p}$ value was corrected to $1.11\sqrt{\alpha }$ (Eq. 8) while the value of $\alpha$ was calculated according to the Eq. 9 from the ${E}_{p}$ and ${E}_{p/2}$ values at each scan rate. Obviously, in reversible processes the factor $L$ is equal to $1$. As it is evident from the Fig. 6, correcting each experimental value of ${I}_{p}$ with the respective $L$ factor is indispensable for the accurate calculation of $A$. Thus, the calculation of $A$ from the slope of this linear plot, over all the examined scan rates, both for the RuHex and for ferricyanide in 0.1. M KCl, pH 3, was conducted and the respective values (0.1015 ± 0.0010 and 0.0951 ± 0.0021 cm^2^) are almost the same with the values calculated from the respective (depending on the $n\Delta {E}_{p}$ at a given scan rate) Randles-Ševčík equations.

### *Heterogeneous electron transfer rate constants (*${k}^{0}$*)*

The same CV experiments used for the calculation of electrode electroactive area with the two different redox probes (RuHex and ferricyanide) can also be exploited for the calculation of heterogeneous electron transfer rate constants.

Regarding the value of $\Delta {E}_{p}$ in each cyclic voltammogram at a specific scan rate, the value of ${k}^{0}$ was calculated either with the Nicholson method ($\Delta {E}_{p}$ < 200 mV) [20] or with the Klingler-Kochi method ($\Delta {E}_{p}$ > 150 mV) [21]. $\Psi$ values (used in Nicholson method) were calculated by Eq. 11 and Eq. 12, for $\Delta {E}_{p}$ < 150 mV and $\Delta {E}_{p}$ > 150 mV, respectively. Specifically, for RuHex the values of ${k}^{0}$ were found (2.535 ± 0.039) × 10^−3^ (Nicholson method), for scan rates 5 – 300 mV s^−1^, and (2.495 ± 0.078) × 10^−3^ cm s^−1^ (Klingler-Kochi method), for scan rates 150 – 500 mV s^−1^. The respective values for ferricyanide in 0.1 M KCl, pH 3 were (1.182 ± 0.150) × 10^−3^ (Nicholson method), for scan rates 5 – 50 mV s^−1^, and (2.197 ± 0.128) × 10^−3^ cm s^−1^ (Klingler-Kochi method), for scan rates 25 – 500 mV s^−1^, while for ferricyanide in 0.1 M PBS, pH 7 the calculation of ${k}^{0}$ is possible only with the Klingler-Kochi method for all scan rates and its value was (0.401 ± 0.022) × 10^−3^ cm s^−1^.

The difference in the ${k}^{0}$ values calculated by the Nicholson method and the Klingler-Kochi method for the case of ferricyanide in 0.1 M KCl, pH 3 can be explained by the fact that the Nicholson method is based on the approximation of the kinetic parameter $\Psi$ for $\alpha$=0.5, without taking into consideration the true value of $\alpha$, especially at high scan rates. Indeed, as stated in the original work by Nicholson, as $\Psi$ becomes sufficiently low (for large $\Delta {\rm E}_{\mathrm{p}}$ values), the error in the calculation of $\Psi$ due to the different values of $\alpha$ (which in this case should not be considered to be 0.5) spans from 5% ($\Psi$=0.5 or $\Delta {\rm E}_{\mathrm{p}}=105 mV)$ to 20% ($\Psi$= 0.1 or $\Delta {\rm E}_{\mathrm{p}}=212 mV)$. On the other hand, the Klingler-Kochi method is based on the fact that the electrochemical system should be forced to the irreversible regime where the influence of $\alpha$ is sound and thus requires the experimental value of $\alpha$ at a given scan rate and considers this value in Eq. 13. For this reason, for $n\Delta {E}_{\mathrm{p}}$ > 150 mV the use of Klingler-Kochi method is more suitable for the calculation of ${k}^{0}$ (as seen in Eq. 13, the value of $\alpha$ is considered), while the Nicholson method remains the best choice for $n\Delta {E}_{\mathrm{p}}$ values up to ca. 140 mV (ideally up to 105 mV) where $\alpha$ can be considered to play a minimum role in the kinetics of the system.

Here it is important to note that contrary to the case of ferricyanide in 0.1 M KCl, pH 3, the two methods gave almost identical results in the case of RuHex in 0.1 M KCl. This is due to the fact that the true values of $\alpha$, as they were found experimentally at each scan rate, were very close to the theoretical value of 0.5.

**Fig. 7:**
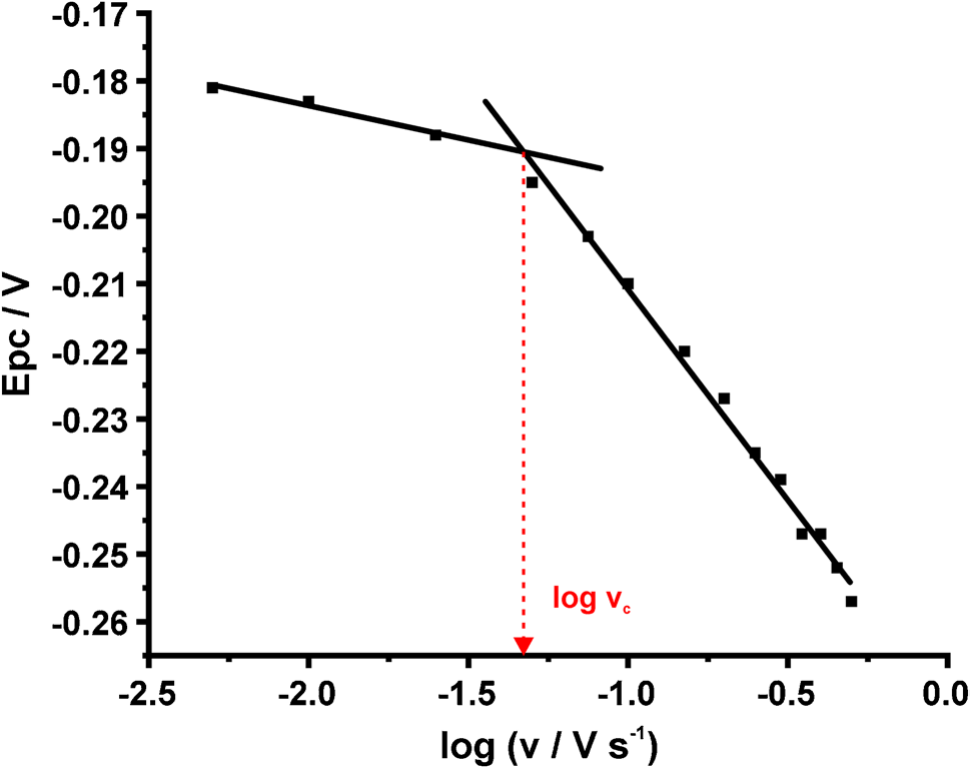
Estimation of ${k}^{0}$ by the Gileadi method. Plot of the peak potential with log of scan rate for the graphical estimation of the critical scan rate (${v}_{c}$). The CVs were conducted in 0.1 M KCl containing 1 mM RuHex at different scan rates

Then, the value of ${k}^{0}$ was estimated with the graphical Gileadi method [22]. This method is not applicable in the case of ferricyanide in 0.1 M PBS, pH 7, because the process is irreversible at every applied scan rate. On the other hand, with the other two examined systems, the estimation of ${k}^{0}$ with the Gileadi method is feasible and the values of ${k}^{0}$ were found to be (3.509 ± 0.143) × 10^−3^ (RuHex) and (3.310 ± 0.250) × 10^−3^ cm s^−1^ (ferricyanide in KCl, pH 3), respectively. At these two cases, due to the transition of the electrochemical process from quasi-reversible to irreversible as the scan rate increases, it is easy to estimate the value of ${v}_{c}$ (i.e., the value where this transition happens). With the same reasoning as for the Klingler-Kochi method, we can deduct that the Gileadi method produces values of ${k}^{0}$ close to that of Klingler-Kochi method because it considers the variation of $\alpha$ (see Eq. 14) both when approaching and after progressing to the irreversible regime. An indicative graph constructed for Gileadi method in the case of RuHex is depicted in Fig. 7.

**Fig. 8:**
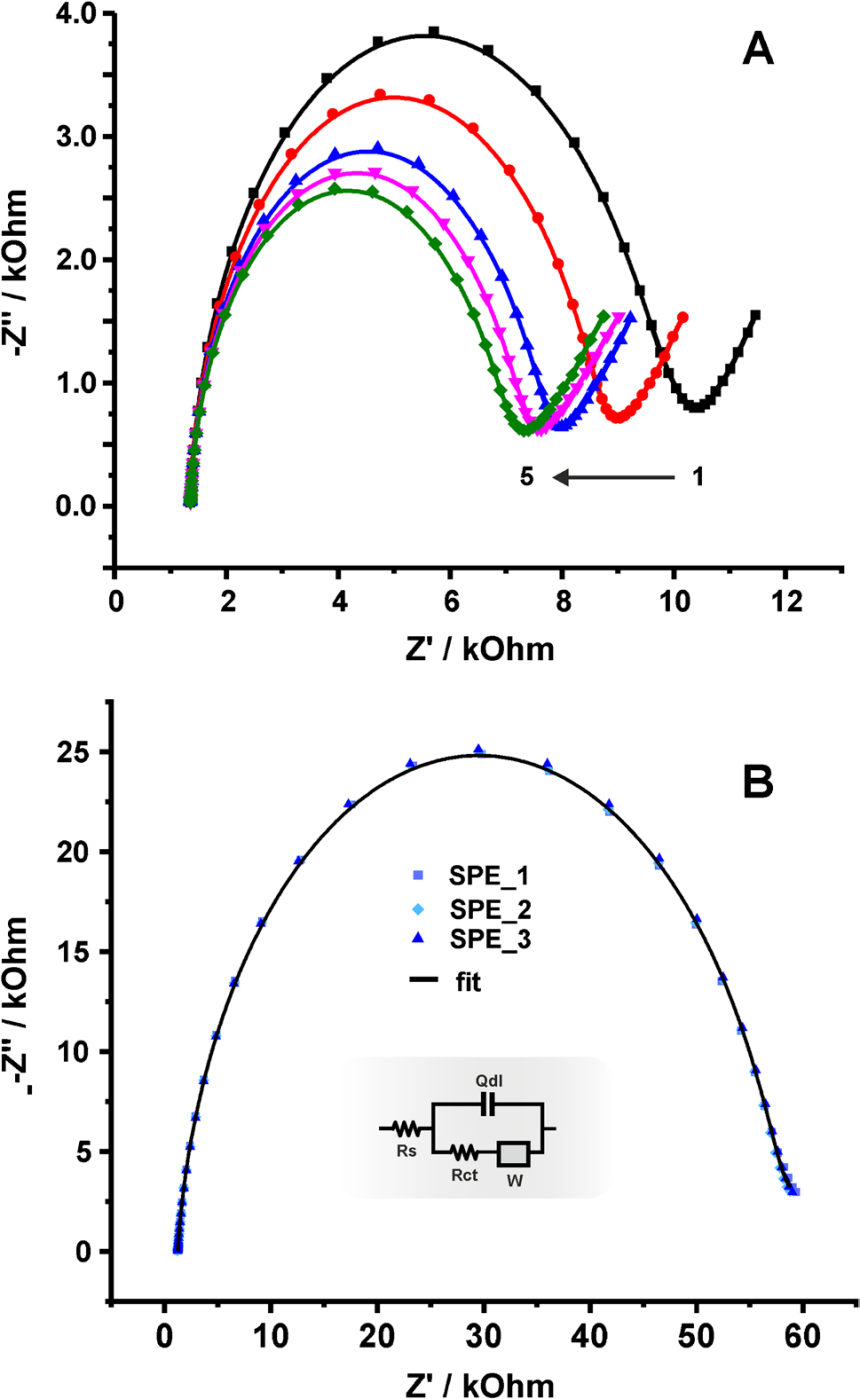
**A** Nyquist plots of successive measurements of a graphite SPE in 0.1 M KCl, pH 3 containing 1 + 1 mM ferro/ferricyanide. **B** (dots) EIS data and (line) fitted curves of 3 successive scans in 0.1 M PBS, pH 7 containing 1 + 1 mM ferro/ferricyanide. Data were modeled to the Randles circuit shown in the inset graph

The estimation of ${k}^{0}$ using EIS data was conducted, as well. The aforementioned ferro/ferricyanide couple in 0.1 M KCl, pH 3 was not the optimum choice due to the system’s instability in successive EIS measurements, as can be seen in Fig. 8A. According to previous studies, this behavior can be attributed to specific adsorption phenomena [31–34]. On the other hand, in 0.1 M PBS pH 7, EIS spectra are quite stable over time. Thus, EIS measurements were conducted in a mixture of 1 + 1 mM ferro/ferricyanide in 0.1 M PBS, pH 7 and the respective spectra (three successive scans) are shown in Fig. 8B. The EIS excitation signal was superimposed to a *dc* potential of 0.210 V (equal to formal potential ${E}^{0}=\left({E}_{pa}+{E}_{pc}\right)/2$, which was found by conducting a CV at 50 mV s^−1^). The composition of the measuring solution and the DC potential were selected to ensure equimolar concentrations of the oxidized and reduced species at the electrode surface at all times (${C}^{*}={C}_{ox}^{*}={C}_{red}^{*}$)_._ EIS data were fitted to a Randles circuit (Fig. 8B, inset), which was used to estimate the value of ${R}_{ct}$ (Table 3) from which (using the Eq. 17) ${k}^{0}$ was estimated (0.022 ± 0.002) × 10^−3^ cm s^−1^.

**Table 3:** Calculated values by fitting the EIS data in Fig. 8B to a Randles circuit

Electrode	${R}_{s}$ (kOhm)	${R}_{ct}$ (kOhm)	${Y}_{0}\times {10}^{6}$ (F cm^−2^ s^n−1^)	$\mathrm{n}$
SPE_a_	1.35	49.2	0.6749	0.927
SPE_b_	1.31	52.1	0.6747	0.928
SPE_c_	1.26	56.5	0.7123	0.922

This value is an order of magnitude lower than the respective value found by the CV-based method for the same system (Table 2). The explanation for this deviation lies on the fact that Eq. 17 is valid for very low overpotential ($\eta$) values as a result of a linear approximation of the Butler-Volmer equation. If no mass-transfer effects are considered, the Butler-Volmer equation is [2]:

18 $$I={i}_{0}[({e}^{-\alpha \left(\frac{F}{RT}\right)\eta }-{e}^{\left[\left(1-a\right)\left(\frac{F}{RT}\right)\eta \right]}]$$

while the approximated linear version of the Butler-Volmer equation is [2]:

19 $$I={-i}_{0}\left(\frac{F}{RT}\right)\eta$$

By definition, $\eta$ is the additional voltage required in a non-reversible electrochemical system (by reference to its formal potential when ${C}_{ox}^{*}={C}_{red}^{*}$) for the electrochemical reaction to occur. For example, $\eta$ for the anodic part of a redox reaction is:

20 $$\eta ={\rm E}_{p\alpha }- {E}^{0}$$

**Fig. 9:**
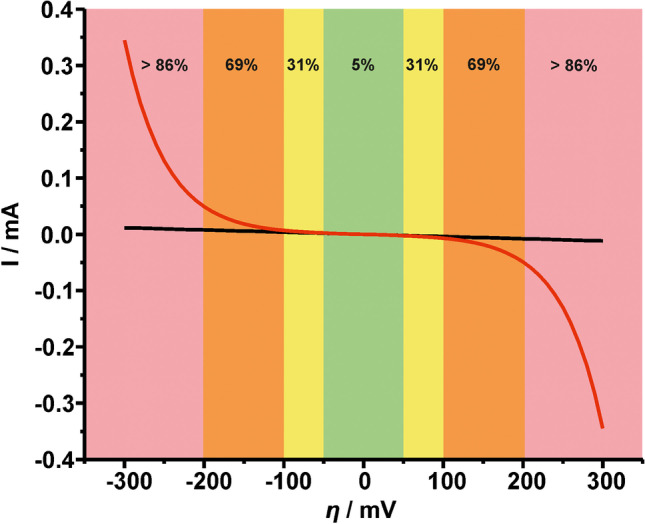
Current-overpotential curves generated by the (red line) Butler-Volmer equation and (black line) its linear approximation at an overpotential range of ± 10 to ± 300 mV. Highlighted areas designate the average current error percentage ${I}_{error}(\%)$; green: 5%, yellow: 31%, orange: 69%, and salmon: > 86%. $\alpha$ was set to 0.5 in the Butler-Volmer equation and ${i}_{0}$ was set to 10^−6^ A in both cases

It is evident that in this specific system (ferro/ferricyanide in 0.1 M PBS, pH 7) the overpotential value is high enough (> 200 mV) to negate the use of Eq. 17 for the calculation of ${k}^{0}$ via EIS measurements since in this case, ${R}_{ct}$ and ${k}^{0}$ are not linearly related. This begs the question about the maximum $\eta$ value that permits the use of Eq. 17 and consequently, the calculation of ${k}^{0}$ via EIS data in a particular electrochemical system (redox molecule, electrolyte, electrode, etc.). In response, we provide a graph illustrating the current values generated by the Butler-Volmer equation (Eq. 18) (${I}_{B.V.}$) and its linear approximation (Eq. 19) (${I}_{L}$) *versus* overpotential (Fig. 9).

The highlighted areas show the average error in current, ${I}_{error}(\%)=[{(I}_{B.V. }-{I}_{L})/{I}_{B.V.}]\times 100$, and consequently the validity of the linear approximation with respect to $\eta$. Within the green area ($\eta$ spans from 0 to ± 50 mV) the mean error is 5%, within the yellow area ($\eta$ spans from ± 50 to ± 100 mV) 31%, within the orange area ($\eta$ spans from ± 100 to ± 200 mV) 69%, and finally, within the salmon area ($\eta >$ ±200 mV) > 86%. With that in mind, we can deduct that a suitable electrochemical system to use for the estimation of ${k}^{0}$ via EIS data would need to have an overpotential value up to 50 mV. We thus suggest, before implementing EIS measurements at a fixed DC potential corresponding to the formal potential of the redox probe in a given electrochemical (electrode, electrolyte) system, the overpotential value to be evaluated by using Eq. 20 by running CV measurements at a medium scan rate (for example, 50 mV s^−1^).

Unlike $A$, which for a single electrode should be identical regardless of the method or the measuring conditions used, comparing each method using ${k}^{0}$ values of different redox probes in different electrolytes is not recommended since ${k}^{0}$ is a system dependent parameter, and is consequently inherently different for each redox probe/electrolyte system. Commenting on both approaches, CV-based methods provide more reliable results in comparison with the EIS-based method, where the validity of the results is dependent on the overpotential value of the examined system. Nonetheless, CV-based methods, while considered to be more reliable, they do need special attention especially in systems traversing between the quasi-reversible to the irreversible regime for all the reasons stated above.

## Conclusions

We have applied and discussed the most widely used techniques and methods reported in literature for the calculation of electrode electroactive area ($\mathrm{A}$) and heterogeneous electron transfer rate constants (${k}^{0}$) using graphite screen-printed electrodes. Considering the factors that should be taken into account when these methodologies are used for non-flat electrode surfaces [11, 13–15], as well as the approximation involved in graphical methods (for example, the Gileadi method), or the use of fitted values from impedance data, the reported results are considered as estimated values. We believe that we have prepared a useful and detailed guide which can instill the proper experimental demeanor in electrochemists.

The electrode electroactive area was calculated using both double-step chronocoulometry (Anson equation) and cyclic voltammetry (Randles-Ševčík equations). Once chronocoulometry with an outer-sphere redox probe (RuHex) produces symmetric and reproducible chronocoulograms and the respective Anson plots Q vs. t^1/2^ are linear with an intercept equals (or it is almost) zero, its use is recommended. On the other hand, when cyclic voltammetry is employed, it is very important the use of the suitable form of the Randles-Ševčík equation with respect to the peak separation value(s) of the cyclic voltammograms at different scan rates. We also introduced “$L$”, a parameter dependent on the system’s behavior (quasi-reversible or irreversible) for the correction of the experimentally obtained peak current values when $A$ is calculated from the slope of a ${I}_{p}=f\left(\sqrt{v}\right)$ plot.

The heterogeneous electron transfer rate constants were calculated using both cyclic voltammetry and electrochemical impedance spectroscopy. Even though cyclic voltammetry- based methods (Nicholson, Klingler-Kochi, and Gileadi) are proved to be more elaborate, they can be applied to every system, considering the $n\Delta {E}_{\mathrm{p}}$ for a given electrochemical system. As a rule of thumb, when $n\Delta {E}_{\mathrm{p}}$>150 mV the use of Klingler-Kochi method is suggested, while the Nicholson method remains the best choice when the $n\Delta {E}_{\mathrm{p}}$ values are lower (ideally up to 105 mV). For an electrochemical system that its cyclic voltammetric behavior at increasing scan rates visibly transits from (quasi)reversible to irreversible regime, the graphical Gileadi method remains also a reliable alternative. On the other hand, electrochemical impedance spectroscopy can provide reliable values for electrochemical systems that exhibit low (typically < 50 mV) overpotentials and their impedance spectra can be modelled to a Randles circuit.
